# Supplementary material for: A Humanized Yeast Phenomic Model of Deoxycytidine Kinase to Predict Genetic Buffering of Nucleoside Analog Cytotoxicity
Source: Genes (Basel). 2019 Sep 30;10(10):770. doi: 10.3390/genes10100770 (PMC6826991; doi:10.3390/genes10100770)
Supplement: Supplementary file 1 [file genes-10-00770-s001.zip › Additional_Files/Additional_File7_GOTermHeatmaps.zip!/Additional_File7_GOTermHeatmaps/nucleolus.pdf]

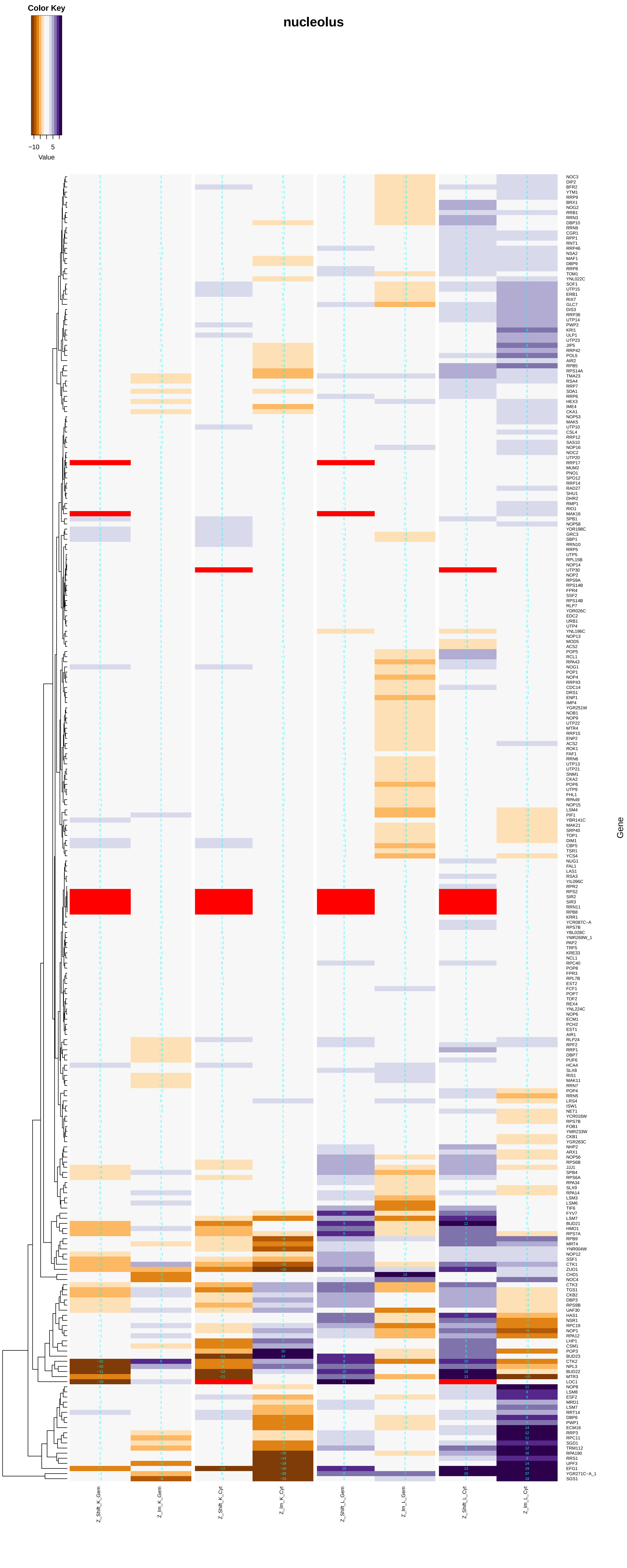

nucleolus
Color Key
−10
5
Value
NOC3
DIP2
BFR2
YTM1
RRP9
BRX1
NOG2
RRB1
RRN3
DBP10
RRN9
CGR1
RPP1
RNT1
RRP46
NSA2
MAF1
DBP9
RRP8
TOM1
YNL022C
SOF1
UTP15
ERB1
RIX7
GLC7
DIS3
RRP36
UTP14
PWP2
KRI1
ULP1
UTP23
JIP5
RRP42
POL5
AIR2
RPB5
RPS14A
TMA23
RSA4
RRP7
SDA1
RRP6
HEX3
IME4
CKA1
NOP53
MAK5
UTP10
CSL4
RRP12
SAS10
NOP16
NOC2
UTP20
RRP17
MUM2
PNO1
SPO12
RRP14
RAD27
SHU1
DHR2
RMP1
RIO1
MAK16
SPB1
NOP58
YDR198C
GRC3
SBP1
RRN10
RRP5
UTP5
RPL15B
NOP14
UTP30
NOP2
RPS9A
RPS14B
FPR4
SSF2
RPS14B
RLP7
YDR026C
EDC2
URB1
UTP4
YNL196C
NOP13
MOD5
ACS2
POP5
RCL1
RPA43
NOG1
POP1
NOP4
RRP43
CDC14
DRS1
ENP1
IMP4
YGR251W
NOB1
NOP9
UTP22
MTR4
RRP15
ENP2
ACS2
ROK1
FAF1
RRN6
UTP13
UTP21
SNM1
CKA2
POP6
UTP9
FHL1
RPA49
NOP15
LSM4
PIF1
YBR141C
MAK21
SRP40
TOP1
DIM1
CBF5
TSR1
YCS4
NUG1
FAL1
LAS1
RSA3
YIL096C
RPR2
RPS2
SIR2
SIR3
RRN11
RPB8
KRR1
YCR087C−A
RPS7B
YBL028C
YMR269W_1
PAP2
TRF5
KRE33
NCL1
RPC40
POP8
FPR3
RPL7B
EST2
FCF1
POP7
TOF2
REX4
YNL224C
NOP6
ECM1
PCH2
EST1
AIR1
RLP24
RPF2
RRP1
DBP7
PUF6
HCA4
SLX8
RIS1
MAK11
RRN7
POP4
RRN5
LRS4
ISW1
NET1
YCR016W
RPS7B
FOB1
YMR233W
CKB1
YGR283C
NHP2
ARX1
NOP56
RPS6B
JJJ1
SPB4
RPS6A
RPA34
SLX9
RPA14
LSM3
LSM6
TIF6
FYV7
LSM7
BUD21
HMO1
RPS7A
RPB9
MRT4
YNR004W
NOP12
SSF1
CTK1
ZUO1
CHD1
NOC4
CTK3
TGS1
CKB2
DBP3
RPS9B
UAF30
HAS1
NSR1
RPC19
NOP1
RPA12
LHP1
CSM1
POP3
BUD23
CTK2
NPL3
BUD22
MTR3
LOC1
NOP8
LSM8
ESF2
MRD1
LSM7
RRT14
DBP6
PWP1
ECM16
RRP3
RPC11
SGD1
TRM112
RPA190
RRS1
UPF3
EFG1
YGR271C−A_1
SGS1
Gene
Z_Shift_K_Gem
Z_Im_K_Gem
Z_Shift_K_Cyt
Z_Im_K_Cyt
Z_Shift_L_Gem
Z_Im_L_Gem
Z_Shift_L_Cyt
Z_Im_L_Cyt
